# The retropharyngeal reduction plate for atlantoaxial dislocation: a finite element analysis

**DOI:** 10.3389/fbioe.2024.1346850

**Published:** 2024-01-22

**Authors:** Weiqing Kong, Yukun Du, Jianyi Li, Jiale Shao, Yongming Xi

**Affiliations:** Department of Spinal Surgery, The Affiliated Hospital of Qingdao University, Qingdao, China

**Keywords:** atlantoaxial dislocation, finite element analysis, retropharyngeal approach, reduction, posterior screw fixation

## Abstract

**Objective:** To investigate the biomechanical properties of the retropharyngeal reduction plate by comparing the traditional posterior pedicle screw-rod fixation by finite element analysis.

**Methods:** Two three-dimensional finite element digital models of the retropharyngeal reduction plate and posterior pedicle screw-rod fixation were constructed and validated based on the DICOM (Digital Imaging and Communications in Medicine) data from C1 to C4. The biomechanical finite element analysis values of two internal fixations were measured and calculated under different conditions, including flexion, extension, bending, and rotation.

**Results:** In addition to the backward extension, there was no significant difference in the maximum von Mises stress between the retropharyngeal reduction plate and posterior pedicle screw fixation under other movement conditions. The retropharyngeal reduction plate has a more uniform distribution under different conditions, such as flexion, extension, bending, and rotation. The stress tolerance of the two internal fixations was basically consistent in flexion, extension, left bending, and right bending.

**Conclusion:** The retropharyngeal reduction plate has a relatively good biomechanical stability without obvious stress concentration under different movement conditions. It shows potential as a fixation option for the treatment of atlantoaxial dislocation.

## Introduction

Atlantoaxial dislocation (AAD) is one of the common diseases in the cranial–cervical junction. In some cases, the mortality rate can reach 35% when severe atlantoaxial dislocation injures the medulla oblongata (Meyer et al., 2019). Studies have shown that AAD caused by traffic injuries accounts for 32.4% of traffic injury deaths, causing significant socioeconomic burden (García-Pallero et al., 2019). A variety of reasons can cause atlantoaxial joint instability, resulting in the loss of the normal alignment of the atlantoaxial articular surface, and then compress the spinal cord, causing numbness, walking instability, paralysis, and even death (Goel, 2019). For improving AAD treatment, some scholars have proposed many classifications, including Fielding, TOI, and Yin (Yin et al., 2005; Xu et al., 2013).

The main treatment for AAD includes conservative treatment (traction and cervical brace) and surgery. Among them, surgery is the most important means of treating AAD. At present, according to the different types of AAD, the surgical treatment methods mainly include anterior release and fixation, posterior reduction fixation, and combined anterior release and posterior fixation (Govindasamy et al., 2020; Unni et al., 2021). For most patients, posterior fixation fusion, with the assistance of skull traction, can achieve good clinical efficacy in one stage (Guo et al., 2020). However, due to the long-term dislocation of the atlantoaxial joint in some patients, the contractures of anterior muscles and ligaments and the anterior hyperosteogeny of the atlantoaxial joint can lead to difficulty in reduction (Zhu et al., 2019). The anterior release is necessary for these conditions in order to better achieve the reduction. At present, the transoral release is the main approach, but it has a relatively high risk of postoperative infection (Dong et al., 2021). In addition, the transoral approach is not suitable for some patients with oral lesions or limited mouth opening.

For those patients, we used the retropharyngeal approach for the atlantoaxial joint release based on clinical experience (Ren et al., 2019). At the same time, we innovatively designed the retropharyngeal reduction plate system inspired by the transoral atlantoaxial reduction plate (TARP). The previous cadaveric simulation experiments showed that the retropharyngeal reduction plate was suitable for placement on the atlantoaxial joint (Li et al., 2022). However, compared with posterior atlantoaxial fixation, there is still a lack of research on the biomechanical properties of the retropharyngeal reduction plate system.

In recent years, the medical finite element analysis method has been widely used in orthopedics (Li et al., 2020; Lewis et al., 2021). By this method, clinicians can conduct preoperative digital simulation of various internal fixation types to obtain the stress and displacement values of different fixation models (Liu et al., 2018). Finite element analysis can provide help for the diagnosis and treatment of clinical orthopedics by selecting the best internal fixation (Rehousek et al., 2018; Wang et al., 2018). Compared with cadaveric experimentation, finite element analysis has obvious advantages in evaluating the biomechanical properties of internal fixation.

Therefore, the aim of this study was to evaluate the biomechanical properties of the retropharyngeal reduction plate and posterior pedicle screw fixation by finite element analysis. The biomechanical stress distribution of the two fixations under different conditions was also measured to provide the theoretical basis for the clinical application of the retropharyngeal reduction plate in the future.

## Materials and methods

### Design of the retropharyngeal reduction plate system

**FIGURE 1:**
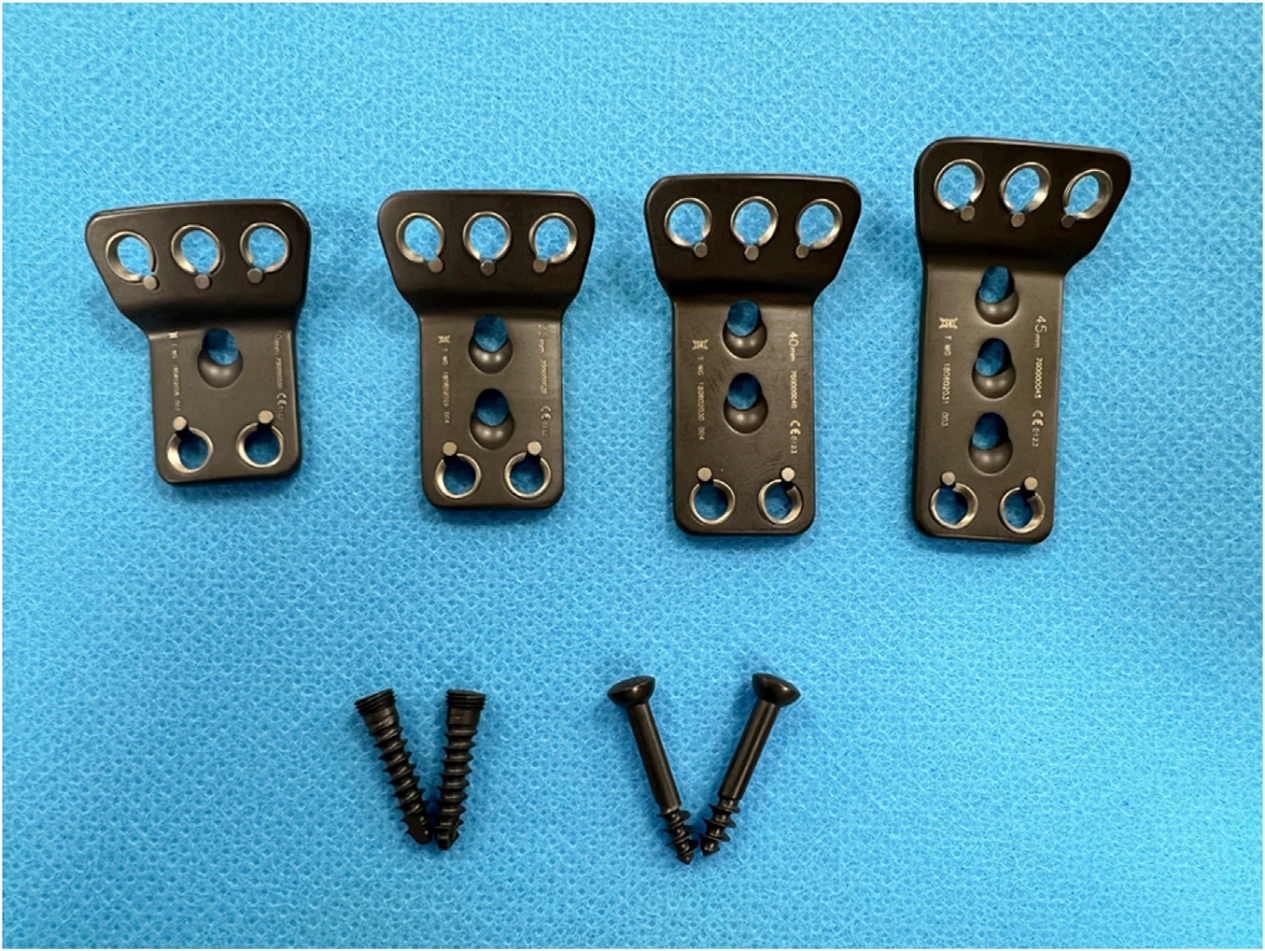
Different sizes of T-type (holes: 6, 7, and 8; length: 25–55 mm; width: 20–25 mm) retropharyngeal reduction titanium plates and the screws (length: 16–18 mm; diameter: 4.0–4.5 mm).

**FIGURE 2:**
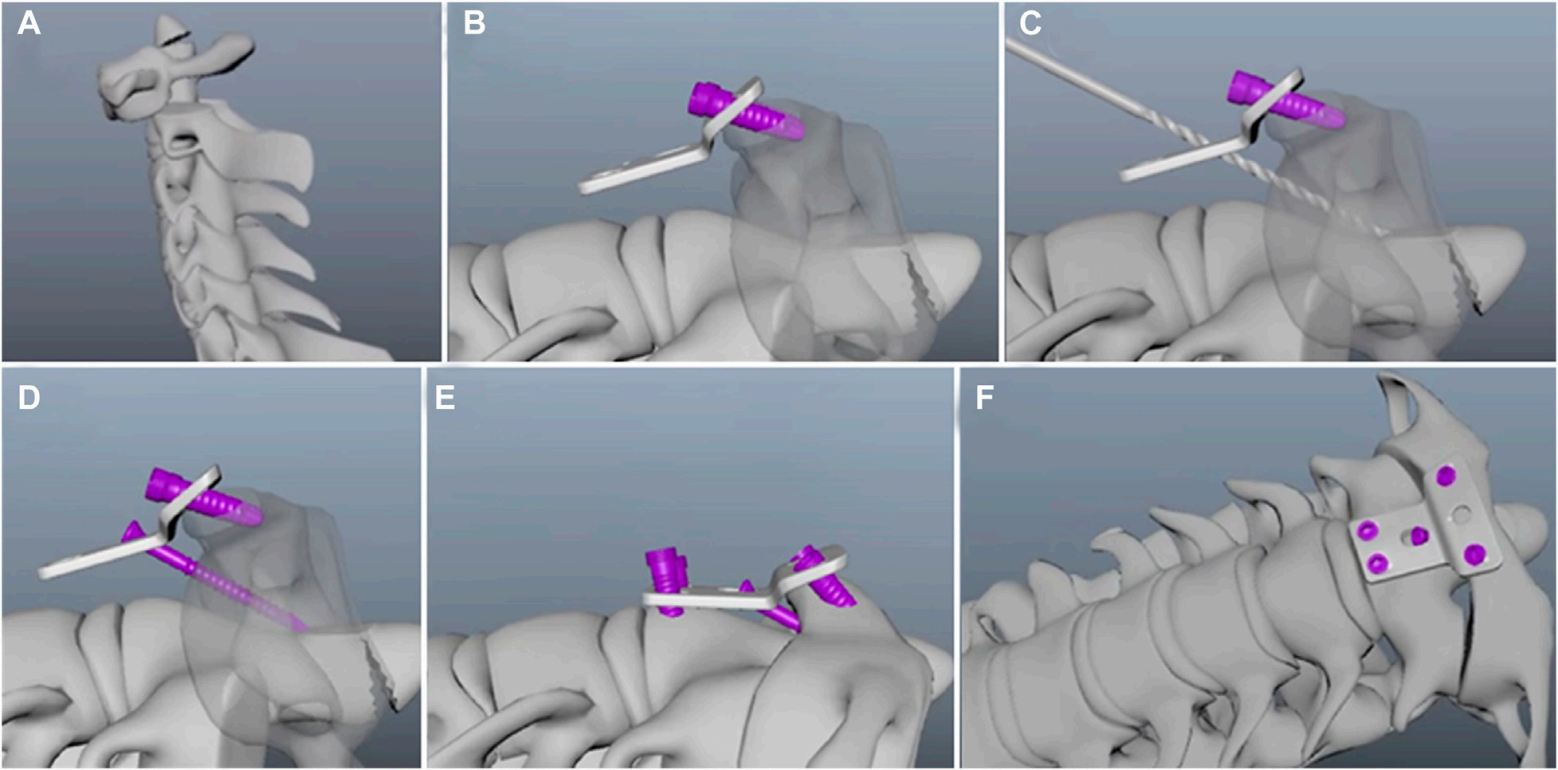
Placement of the retropharyngeal reduction plate. **(A)** Atlantoaxial dislocation. **(B)** Fixation on the C1 vertebral body by inserting screws through upper round holes. **(C)** Construction of the lag screw path through an odontoid process using a hand drill. **(D)** Lag screw insertion through the special oval hole to achieve reduction. **(E)** Fixation on the basal part of the C2 vertebral body. **(F)** Placement of the retropharyngeal reduction plate on the atlantoaxial joint.

The retropharyngeal reduction plate system is characteristic of T-type titanium plates, which includes the screws and different sizes of plates (GB4Z180193509, WEGO ORTHO Co., Ltd.) (Li et al., 2022). The specific plate angle (30°–35°) of horizontal and vertical parts can be conducive to the placement onto the atlantoaxial joint. The number of round holes (diameter: 4.5–5.0 mm), used for inserting screws (diameter: 4.0–4.5 mm and length: 16–18 mm), depends on the size of the plates. The length and width of the plates range from 25 to 55 mm and 20 to 25 mm. In addition, the specially designed oval holes (diameter: 5.0–5.5 mm), in the center of the plates, are designed for facilitating reduction by inserting lag screws into the axis. After the anterior release of the atlantoaxial joint, the retropharyngeal reduction plate can achieve reduction by inserting the lag screws to provide the atlantoaxial joint with forward and downward tractions in one stage (Figures 1, 2).

### The development of finite element models

Cadaveric specimens with atlantoaxial dislocation who had undergone surgical treatment using the retropharyngeal reduction plate were included in this study. The retropharyngeal reduction plate was placed on the atlantoaxial joint of cadaveric specimens. A 45-year-old female atlantoaxial dislocation patient who had undergone the posterior pedicle screw-rod fixation was enrolled in this study. The patient was informed of the experimental procedures and gave written informed consent for participation in this study. Spiral CT scanning was performed from the base of the occipital bone to the C7 vertebrae using 0.5-mm-thick slices. The scanning images were collected and stored in DICOM format for three-dimensional reconstructions using MIMICS 20.0 software (Materialise, Leuven, Belgium). A rough geometric model of cervical vertebrae was obtained by conducting commands, including threshold segmentation and regional growth.

**FIGURE 3:**
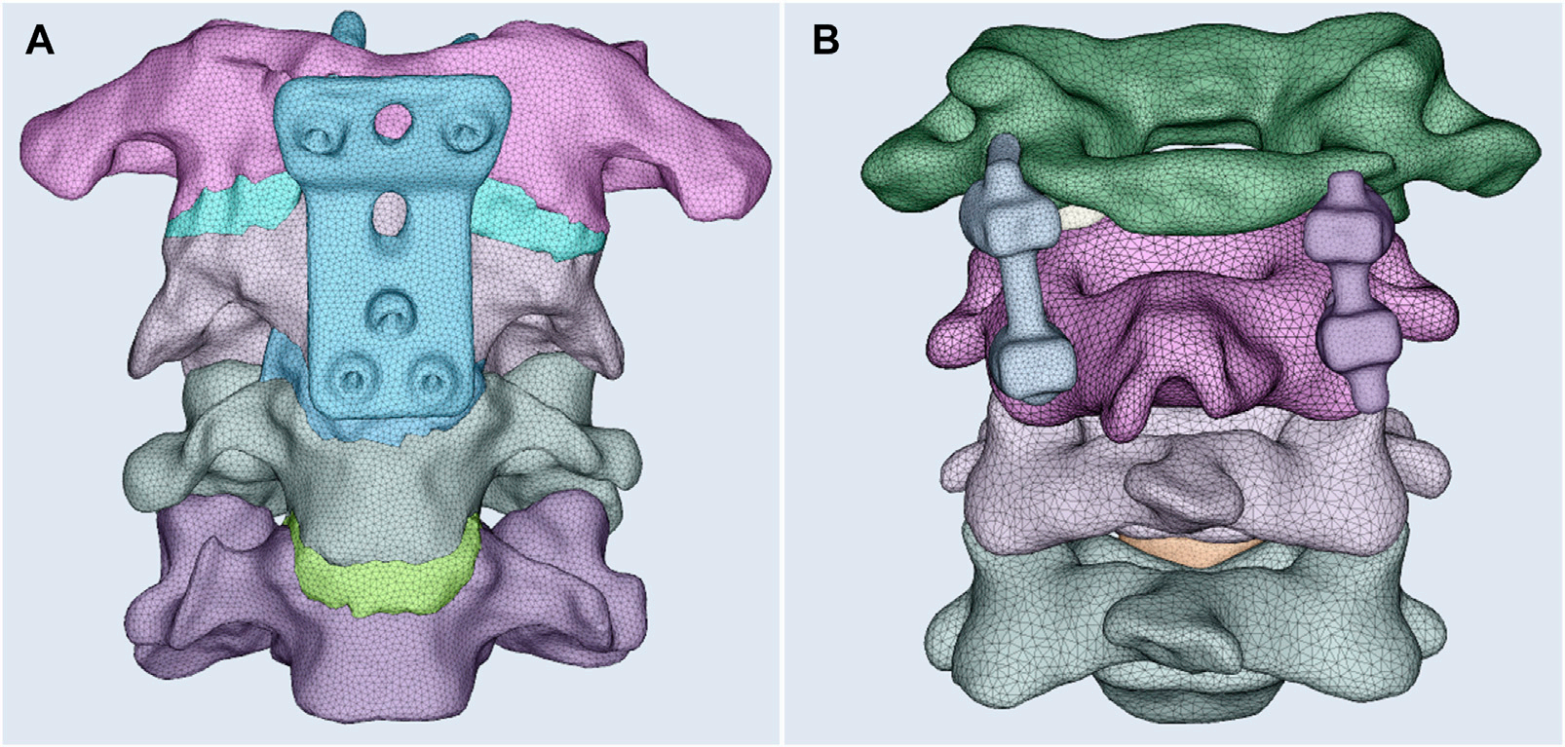
Three-dimensional finite element model of the retropharyngeal reduction plate **(A)** and the posterior pedicle screw-rod fixation **(B)**.

After three-dimensional reconstruction, the stereolithography file was imported into 3-matic 10.0 (Materialise, Leuven, Belgium) to further optimize the model for finite element analysis. For the accuracy of finite element analysis, 3-matic 10.0 (Materialise, Leuven, Belgium) was used to construct a geometric solid model of the bone, cartilage, and intervertebral discs by a series of software commands, including sanding, filling, and denoising processes. After obtaining the model, the thin-walled characteristics and curvature of the model were analyzed to understand the basic structure of the atlantoaxial joint model. In order to ensure that the model has no geometric defects, the repair of the atlantoaxial joint model was managed using 3-matic 10.0 (Materialise, Leuven, Belgium). The model consisted of C1–C4 cervical vertebrae and three intervertebral discs (Figure 3). The mesh and material properties were analyzed and assigned for further analysis using HyperMesh 2018 (Altair Engineering, Inc., Troy, Michigan, United States of America).

### The finite element analysis of the two fixation models

The finite element analysis was achieved using HyperWorks 2019 (United States of America, Altair). Based on the parameters of Young’s modulus and Poisson’s ratio, the scientific assignment of elements was obtained (Wang et al., 2019; Chen et al., 2020). The Young’s modulus and Poisson’s ratio values of different structures are shown in Table 1. For achieving the simulation of the stress to the cervical vertebra from the head due to gravity, 45 newtons (N) of vertical downward pressure was imposed on the surface of the occipital condyle. Approximately 1.5 Nm torque was imposed on the models from different directions to simulate the load of cervical vertebrae under normal physiologic conditions (Bo et al., 2016). We measured and calculated the stress of the two internal fixations under different conditions, including flexion, extension, bending, and rotation.

**TABLE 1:** Material property of the atlantoaxial joint models.

Element	Young’s modulus (MPa)	Poisson’s ratio
Cancellous bone	450	0.23
Cortical bone	10,000	0.3
Endplate	500	0.4
Spinous process	3,500	0.25
Facet	10	0.4
Nucleus pulposus	1	0.49
Annulus fibrosus	110	0.3

The mechanical properties of the two different internal fixations were comprehensively analyzed, including the 1) maximum von Mises stress of the fixation; 2) stress distribution of different fixations under six conditions; and 3) stress distribution of the plate, screws, and rods.

## Results

### The establishment of the two fixation models

The suitable retropharyngeal reduction plate was located at the body of the atlantoaxial joint by inserting screws. The posterior pedicle screw fixation model was obtained after assembling to simulate the posterior surgery. The satisfying finite element models of the two different fixations were obtained after smoothing and remeshing finite element mesh generation (Figure 3), respectively.

### The validation of the two fixation models

**FIGURE 4:**
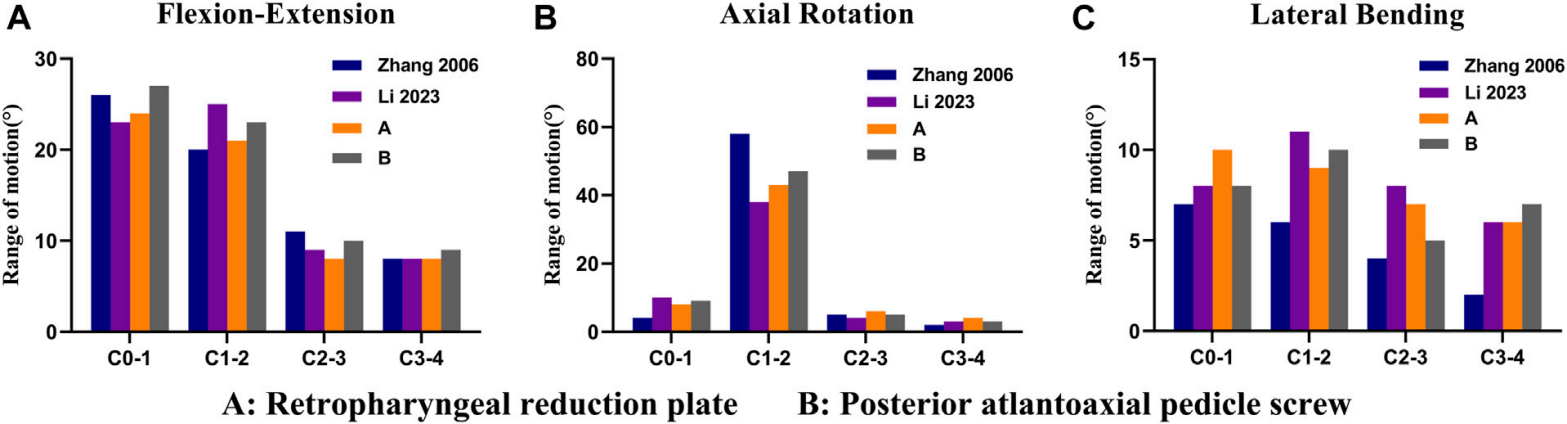
Validation of the two fixation models under a pure moment of 1.5 Nm. Flexion–extension **(A)**, axial rotation **(B)**, and lateral bending **(C)**.

The range of motion (ROM) values of the two fixation models were compared with previous studies by (Figure 4) Zhang et al. (2006) and Li et al. (2023), respectively. Under different movements, the results are relatively consistent with previous studies. Hence, the two fixation models were reliable for further finite element analysis.

### The stress analysis of the two fixation models

**FIGURE 5:**
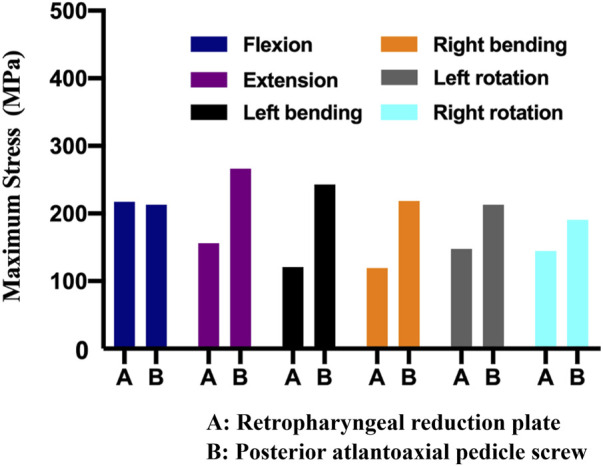
Maximum von Mises stress of the two different fixations under different movements, including flexion, extension, bending, and rotation.

The maximum von Mises stress values of the retropharyngeal reduction plate and posterior pedicle screw fixation were 217.2 MPa and 213 MPa under forward flexion, respectively. However, the maximum von Mises stress of the retropharyngeal reduction plate was significantly lower than that of the posterior pedicle screw fixation during the backward extension (156 MPa vs. 266.3 MPa). The maximum von Mises stress values of the left bending and right bending of the retropharyngeal reduction plate were 120.8 MPa and 119.2 MPa, respectively. The maximum von Mises stress values of the left bending and right bending of the posterior pedicle screw fixation were 242.7 MPa and 218.4 MPa, respectively. Under rotation, the maximum von Mises stress values of the retropharyngeal reduction plate were 147.7 MPa (left) and 144.5 MPa (right), respectively. The maximum von Mises stress values of the posterior pedicle screw fixation were 213.1 MPa (left) and 190.5 MPa (right) under rotation (Figure 5), respectively.

### The stress distribution of the two internal fixation models

**FIGURE 6:**
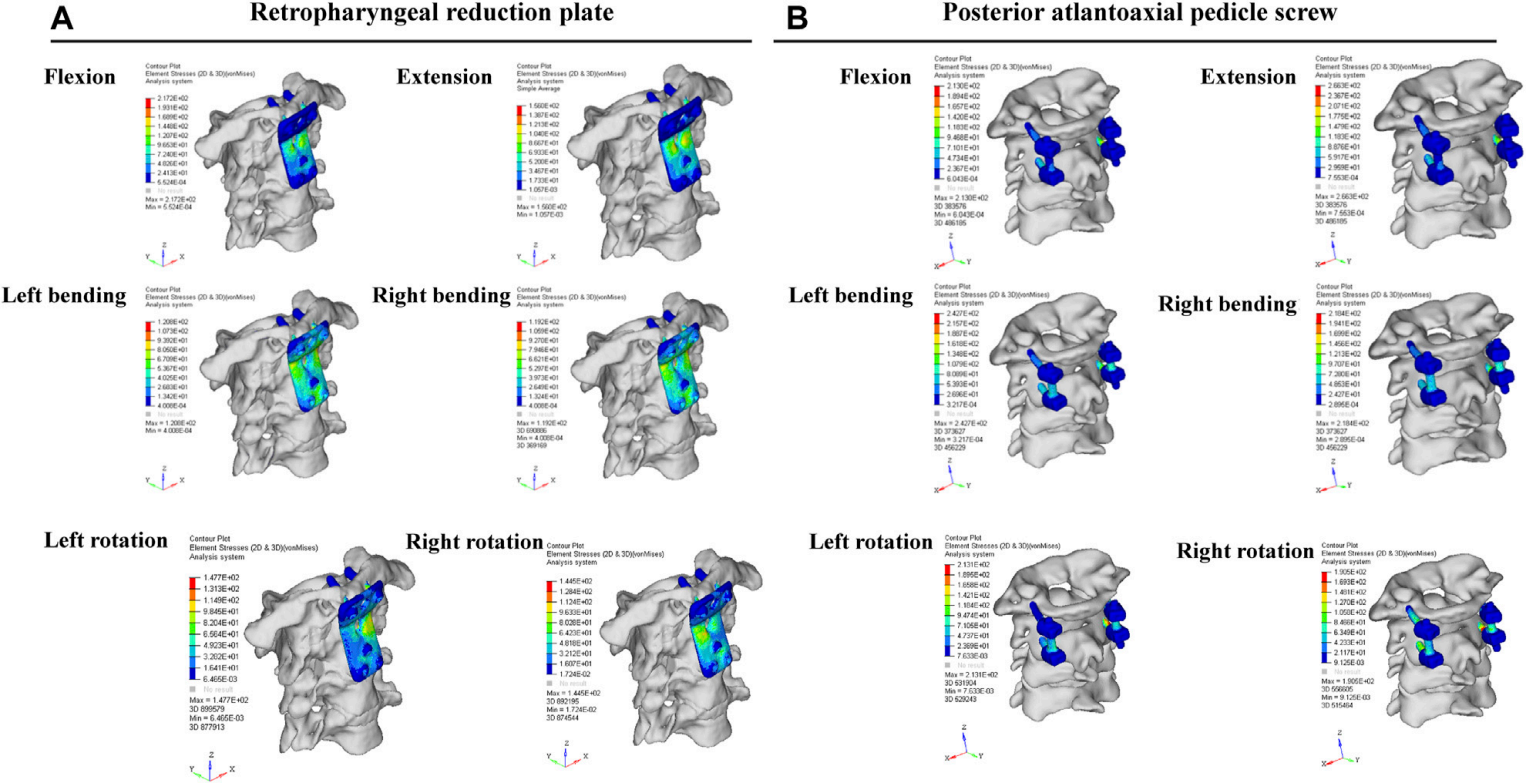
Finite element analysis results of the retropharyngeal reduction plate and posterior pedicle screw-rod fixation under different movements, including flexion, extension, bending, and rotation.

The redness represents the maximum element stress, while the minimum element stress is shown by the blueness. The more uniform distribution of the retropharyngeal reduction plate was noticed under different conditions, such as flexion, extension, bending, and rotation. As shown in Figure 6, the stress was mainly distributed in the lower half of the plate under different conditions, and there was no significant stress concentration in the plate and screws. In the posterior pedicle screw fixation, the junctions of the screw, bone, and rod have relatively more stress in flexion, extension, bending, and rotation. The stress tolerance of the two internal fixations was basically consistent in flexion, extension, left bending, and right bending. In addition, the mean stress of the two internal fixations was far below the maximum yield strength (795–827 MPa) and ultimate strength (860–896 MPa) of titanium alloy.

## Discussion

For some severe AAD patients with obvious neck pain, limb weakness, and trunk and limb numbness, surgery is the appropriate treatment with the purpose of achieving atlantoaxial reduction to maintain the stability of the atlantoaxial joint and to achieve spinal cord decompression to alleviate clinical symptoms (Song et al., 2017; García-Pallero et al., 2019; Goel, 2019). At present, the posterior atlantoaxial fusion is the effective surgical method for the treatment of AAD. Since the 20th century, many scholars have proposed a variety of internal fixation methods for AAD, including cable, lamina hook, and screw-rod internal fixation (Ren et al., 2019; Zhu et al., 2019). In addition, many alternative technologies have emerged for different conditions of AAD patients, such as Magerl screw, C1 lateral mass screw, and C2 lamina screw (Kenzaka, 2019).

With the in-depth study on the treatment of AAD, some scholars found that simple posterior internal fixation and fusion cannot achieve good clinical reduction for patients with irreducible atlantoaxial dislocation due to the anterior bone fusion of the atlanto-odontoid joint or scar contracture of surrounding ligaments and muscles (Ren et al., 2019; Zou et al., 2020). In this condition, the anterior release is necessary to achieve a better atlantoaxial reduction before the atlantoaxial internal fixation.

For now, the anterior release can be achieved by the transoral, endoscopic endonasal, or retropharyngeal approach (Zhu et al., 2019). Wang et al. first used one-stage transoral release reduction combined with posterior internal fixation and fusion, with a 71.9% effectivity rate, in the treatment of irreducible AAD (Wang et al., 2006). The transoral release is a common surgical approach for AAD treatment with the advantage of directly exposing the ventral structure of the craniocervical junction to help the release of the atlantoaxial joint under direct vision. In addition, some scholars have also proposed the concept of transoral release combined with the fixation method in one stage and designed corresponding internal fixation instruments, such as the Harms plate and TARP (Yin et al., 2005; Zhu et al., 2019). However, there are some inevitable limitations to the transoral approach, such as postoperative infection, dysphagia, and laryngeal edema. The transoral approach often requires tracheotomy before operation and postoperative nasal feeding for a long time, which makes postoperative nursing difficult (Amelot et al., 2018).

The retropharyngeal approach is relatively safe because of few surrounding important blood vessels and nerves in this area. The retropharyngeal approach not only can fully expose bilateral atlantoaxial lateral mass joints but also has a low risk of complications, including infection and laryngeal edema (Alshafai and Gunness, 2019). However, there are few reports on internal fixation based on the retropharyngeal approach. Inspired by the TARP, we newly designed the retropharyngeal reduction plate system based on clinical experience. The retropharyngeal reduction plate system can achieve one-stage reduction and fixation after the anterior release (Li et al., 2022). However, the difference in internal fixation strength and stress distribution between the retropharyngeal reduction plate and posterior pedicle screw fixation under different movements is still unknown.

In recent years, the finite element analysis is widely used in the application of orthopedic biomechanics (Taylor and Prendergast, 2015; Bo et al., 2016; Wang et al., 2018). The three-dimensional model is able to be reconstructed based on the DICOM data using the three-dimensional modeling software program. The various surgical conditions can be numerically simulated by finite element analysis (Ansys, Abaqus, or HyperMesh) (Li et al., 2020). Finally, the stress and displacement values of the stress model are obtained, which provides a reference basis for clinical diagnosis. Finite element analysis can analyze the stress distribution of adjacent vertebral bodies, intervertebral discs, and other structures. Chen et al. constructed the complete three-dimensional cervical spine model from C4 to C7 and simulated three different surgical methods, namely, percutaneous full-endoscopic anterior cervical discectomy (PEACD), posterior cervical foraminotomy (PCF), and anterior cervical discectomy and fusion (ACDF) (Chen et al., 2020). They also studied the range of motion, intervertebral disc pressure, and facet joint contact force between surgical segments and adjacent segments under different movement conditions using finite element analysis software. The results showed that the PCF and PEACD methods are more suitable for the treatment of cervical spondylotic radiculopathy compared to ACDF from the perspective of biomechanics, and PCF may be better than PEACD. In addition, the finite element analysis is also able to simulate the clinical application of the new internal fixation technique and analyze the stress distribution of internal fixation under different conditions in order to evaluate the clinical application value of the new internal fixation technique (Lewis et al., 2021). Yu et al. designed two thoracic interbody fusion cages according to the CT anatomical parameters of 150 patients, and the stability of the two interbody fusion cages was compared with the assistance of three-dimensional finite element analysis (Yu et al., 2020). The results showed that both kinds of interbody fusion cages had good biomechanical stability, and the stability of the kidney-shaped fusion cage was better than that of the box-shaped fusion cage.

In this study, we designed the retropharyngeal reduction plate based on a large number of previous clinical experiences to achieve atlantoaxial reduction and fixation in one stage (Ren et al., 2019; Guo et al., 2020; Zou et al., 2020). The previous cadaveric simulation experiments proved that the retropharyngeal reduction plate could be successfully fixed in front of the atlantoaxial joint through the retropharyngeal approach (Li et al., 2022). Aiming to analyze the fixation strength and stress distribution of the retropharyngeal reduction plate, compared with the posterior pedicle screw fixation, the three-dimensional finite element analysis was conducted. The results also showed that the maximum stress of the retropharyngeal reduction plate was significantly lower than that of the posterior pedicle screw fixation (156 MPa vs. 266 MPa) under the backward extension. Under other movement conditions, the stress distribution of the retropharyngeal reduction plate is not significantly different compared with that of the posterior pedicle screw fixation. In addition, the stress was mainly distributed in the lower half of the plate but with no obvious stress concentration in the plate and screw.

This study still has several limitations. First, the finite element modeling process relatively simplified the motion of the spine, and the real motion state of the cervical spine may be more complex. Second, finite element modeling is not able to fully simulate the complex surrounding ligaments and muscles of the atlantoaxial joint.

## Conclusion

In conclusion, the retropharyngeal reduction plate showed good biomechanical stability under different movement conditions, and the stress distribution was more uniform without obvious stress concentration. In the future, the application of the retropharyngeal reduction plate in the clinic is expected to be regarded as one of the treatment methods for AAD.

## Data Availability

The original contributions presented in the study are included in the article/Supplementary Material; further inquiries can be directed to the corresponding author.

## Abbreviations

AAD, atlantoaxial dislocation; TARP, transoral atlantoaxial reduction plate; PEACD, percutaneous full-endoscopic anterior cervical discectomy; PCF, posterior cervical foraminotomy; ACDF, anterior cervical discectomy and fusion.
